# *IQSEC2*-related encephalopathy in male children: Novel mutations and phenotypes

**DOI:** 10.3389/fnmol.2022.984776

**Published:** 2022-10-03

**Authors:** Xinting Liu, Shan Zhang, Lin Wan, Xiaoli Zhang, Haiping Wang, Hongwei Zhang, Gang Zhu, Yan Liang, Huimin Yan, Bo Zhang, Guang Yang

**Affiliations:** ^1^Department of Pediatrics, The First Medical Center, Chinese PLA General Hospital, Beijing, China; ^2^Senior Department of Pediatrics, The Seventh Medical Center of PLA General Hospital, Beijing, China; ^3^Medical School of Chinese PLA, Beijing, China; ^4^Fuxing Road Clinic, Jingnan Medical District, Chinese PLA General Hospital, Beijing, China; ^5^Department of Pediatrics, The Third Affiliated Hospital of Zhengzhou University, Zhengzhou, China; ^6^Department of Pediatric Neurology, Hangzhou Children's Hospital, Hangzhou, China; ^7^Department of Neurology, Jinan Children's Hospital, Jinan, China; ^8^Department of Neurology, Boston Children's Hospital, Harvard Medical School, Boston, MA, United States; ^9^Biostatistics and Research Design Center, Institutional Centers for Clinical and Translational Research, Boston Children's Hospital, Harvard Medical School, Boston, MA, United States; ^10^The Second School of Clinical Medicine, Southern Medical University, Guangzhou, China

**Keywords:** *IQSEC2* gene, trio WES, global developmental delay, seizure, inactivation of the X chromosome

## Abstract

The isoleucine–glutamine (IQ) motif and Sec7 domain-containing protein 2 (*IQSEC2)* gene, located at Xp11. 2, are associated with nervous system diseases, such as epilepsy, autism, and intellectual disabilities. Gender-related differences in the severity of phenotype severity have been described previously. Here, we report the details of seven male children with *IQSEC2* mutations from different families. During this investigation, we explored the relationship between the genotype and phenotype of *IQSEC2* mutations; to do so, we recruited seven children with pathogenic/likely pathogenic *IQSEC2* mutations who were diagnosed with global developmental delay and/or epilepsy. Their clinical features were assessed, and Trio-based whole-exome sequencing (trio WES) was conducted in seven pedigrees. A variety of algorithms and computational tools were used to calculate the pathogenicity, protein stability, conservation, side chain properties, and protein-protein interactions of mutated proteins. The seven patients ranged in age from 18 months to 5 years. Among them, six children were found to have both developmental delay and epilepsy, and one child only exhibited developmental delay. Four novel mutations (c.316C > T, c.443_4 44dup, c.3235T > C, and c.1417G > T) were newly reported. Two patients did not have truncated aberrant proteins caused by missense mutations. Still, they did have severe phenotypes, such as early-onset epilepsy in infancy, because the mutations were located in domains like the pleckstrin homology and IQ calmodulin-binding motif domains. The bioinformatics analysis also proved that missense mutations may be located in the functional region, which affects protein stability and is harmful. In summary, severe phenotypes, such as early-onset epilepsy in infancy, occur in male patients with a missense mutation in specific domains (e.g., pleckstrin homology and IQ calmodulin-binding motif domains). Some female individuals with *IQSEC2* mutations may be asymptomatic because of the skewed inactivation of the X chromosome.

## Introduction

The isoleucine–glutamine (IQ) motif and Sec7 domain-containing protein 2 (*IQSEC2*) gene is associated with neurologic disorders, including intellectual disability (ID), epilepsy, and autism (Hinze et al., 2017; Levy et al., 2019; Mignot et al., 2019; Shoubridge et al., 2019; Wayhelova et al., 2020). *IQSEC2* encodes guanine nucleotide exchange factors (GEFs) for the adenosine diphosphate ribosylation factor (ARF) family of GTP-binding proteins (Shoubridge et al., 2010a). It is an X-linked gene located at Xp11.2. The Sec7 domain in the IQSEC2 protein catalyzes the GDP–GTP exchange on the ARF superfamily of small GTPases (Briševac et al., 2021). The IQSEC2 protein is mainly expressed in the brain. It plays an essential role in modulating the cytoskeleton and vesicle transport at the post-synaptic density, which is a crucial modifier of synaptic plasticity (Murphy et al., 2006).

Previous studies have shown that female and male individuals carrying the same *IQSEC2* mutations may present with different phenotypes because the *IQSEC2* gene escapes X chromosome inactivation in females (Li and Carrel, 2008; Shoubridge et al., 2010a). The mutated *IQSEC2* gene is partially expressed by the escape of X chromosome inactivation. Thus, IDs are less severe in females than in males (Mignot et al., 2019). Moreover, male patients show more severe ID and higher epilepsy incidence rates. Some female patients carrying missense mutations in *IQSEC2* have asymptomatic or mild ID (Shoubridge et al., 2010a, 2019; Kalscheuer et al., 2015). Compared to males, heterozygous female carriers of pathogenic *IQSEC2* variants typically manifest phenotypes in milder forms (Shoubridge et al., 2019). As for mutation types, Tran Mau-Them et al. (2014) proposed that truncating mutations (e.g., nonsense, frameshift) in *IQSEC2* can cause a loss of function and severe ID. However, Shoubridge et al. (2022) indicated that missense variants in certain domains like the pleckstrin homology (PH) domain might also generate seizures and speech and language deficits.

To reveal the characteristics of the disease in male patients and whether only truncating mutations will lead to severe phenotypes or not, we herein describe the genotypes and clinical phenotypes of seven male children from different families with *IQSEC2* mutations, including five *de novo* mutations and two maternal inheritances. In addition, bioinformatic analysis was conducted to predict whether the mutations will affect the structure and function of the IQSEC2 protein or not. In short, we sought to explore and explain the relationship between the genotype and phenotype of the *IQSEC2* mutation in male children.

## Patients and methods

### Patients

Seven patients with confirmed *IQSEC2* mutations who were diagnosed with global developmental delay and/or epilepsy were recruited from the First Medical Center of PLA General Hospital, the Third Affiliated Hospital of Zhengzhou University, and Hangzhou Children's Hospital, and Jinan Children's Hospital. Detailed clinical information was collected, such as seizure type, clinical manifestations, Electroencephalogram (EEG), and magnetic resonance imaging (MRI) findings. Genomic DNA was extracted from the peripheral blood leukocytes of patients and their parents for trio-based whole-exome sequencing (Trio-WES). Each patient's guardians provided informed consent, and the study was approved by the ethics committee of the First Medical Center of the PLA General Hospital.

### Trio-based whole-exome sequencing

Trio-WES was performed for probands and their parents. Briefly, venous blood was extracted from all probands and their parents into EDTA anticoagulant tubes. Next, genomic DNA was extracted from venous blood samples using a RelaxGene Blood DNA system (Tiangen Biotech Co., Ltd., Beijing China). The libraries for WES were constructed using a NanoPrep DNA Library Preparation Module (for MGI), with 96 reactions. Following this, the libraries were sequenced on a BGI MGISEQ-2000 sequencer in 2 × 150-bp paired-end reads at a minimum of 150 × coverage. After obtaining the raw reads, read alignment was performed using the Burrows–Wheeler Aligner tool (version 0.7.17) with default parameters against the human genome assembly hg19 (GRCh37). The generated bam file was sorted and deduplicated using SAMtools and Picard. Next, the Genome Analysis Toolkit (GATK, https://software.broadinstitute.org/gatk/) was applied to detect single-nucleotide variants (SNVs) and indels (<50 bp). The CNVkit was used to detect the copy number variations (CNVs). The 1,000 Genome Project, Genome Aggregation Database, and Exome Aggregation Consortium were employed to annotate the frequency of the variants in the population. Additionally, the Online Mendelian Inheritance in Man (OMIM), Human Gene Mutations database (HGMD), and ClinVar database were employed to annotate related diseases. All variants were evaluated for possible side effects on protein function by three widely used prediction tools, Polymorphism Phenotyping version 2 (PolyPhen-2), Sorting Intolerant from Tolerant (SIFT), and MutationTaster, SpliceAI. Two missense mutations (c.3235T > C, p.Ser1079Pro; c.1075C > T, p.Arg359Cys) were further analyzed by the mean of bioinformatics for the pathogenicity. The stability, conservation, side chain properties and protein-protein interactions of IQSEC2 protein were studied. The process will be described in detail in the third part of Methods. The candidate pathogenic variants associated with disease phenotypes were obtained according to American College of Medical Genetics and Genomics (ACMG) guidelines.

*IQSEC2* mutations were verified by Sanger sequencing. Primers were designed for suspected pathogenic sites, and the corresponding genomic DNA regions were subjected to a polymerase chain reaction (PCR). Products were separated with 0.8% agarose gel electrophoresis and purified using a QIAquick gel extraction kit (Qiagen, Hilden, Germany). Subsequently, the effects were sequenced using the 3730 x l DNA analyzer sequencing standards, BigDye™ Terminator version 3.1 (Thermo Fisher Scientific, Waltham, MA, USA), and 3730 x l DNA analyzer (Thermo Fisher Scientific).

### Bioinformatic analyses

#### *IQSEC2* protein information

The amino acid sequence of the IQSEC2 protein in FASTA format was acquired from the National Center for Biotechnology Information (https://www.ncbi.nlm.nih.gov/homologene). IQSEC2 protein has no complete protein structure data in Protein Data Bank (PDB) format (protein name: 6FAE), and the prediction of full-length protein data as Alphafold was supported by the UniProt database (https://www.uniprot.org) (UniProt ID: Q5JU85).

#### Pathogenicity and deleterious analysis

The following tools were used to predict the pathogenicity of missense mutations: Combined Annotation-Dependent Depletion (CADD), PolyPhen, SIFT, and MutPred. CADD is a computational tool designed to predict the deleteriousness of variants (Rentzsch et al., 2019). It is generally believed that the mutation is disease-causing if the CADD value is >20 (Shyr et al., 2014; Halvardson et al., 2016). PolyPhen-2 web server calculates the likelihood of disease-related amino acid substitution. Scores ranging from 0 to 1 point(s) may be interpreted as probably damaging, possibly damaging, or benign (Adzhubei et al., 2013; Saih et al., 2021a). SIFT aims to distinguish the intolerant or tolerant amino acid changes. It was carried out to predict amino acid changes which affect protein function. The substitution is considered deleterious when the SIFT value is <0.05 (Levy et al., 2019). MutPred, which can infer the molecular cause and phenotypic impact of amino acid alterations, provides the results of pathogenicity prediction and molecular changes possibly influencing the phenotype (Pejaver et al., 2020).

#### Protein stability analysis

A single amino acid mutation can produce a free energy change after folding called ΔG or DG, which can change the structural stability of the protein. We calculated the differences in folding free energy between wild-type and mutant proteins (ΔΔG or DDG) to evaluate the effect of mutation on protein stability (Laskowski et al., 2020). iStable (http://predictor.nchu.edu.tw/istable/indexSeq.php) was used to construct a sequence-based model and predict the stability change due to single-site mutations (Chen et al., 2020). CUPSAT (http://cupsat.tu-bs.de/) is a bioinformatics tool that predicts protein stability changes combined with torsion angle potentials and structural environment–specific atom potentials (Saih et al., 2021b). The value of ΔΔ G classifies the prediction of the stability trend into two types: a decrease in stability (<0 kcal/mol) and an increase in stability (>0 kcal/mol) (Chen et al., 2013; Saih et al., 2021b). MUpro (http://mupro.proteomics.ics.uci.edu/) is a software program for predicting the stability change of mutated protein structures. DUET uses a support vector machine to estimate the value and direction of the free energy stability (Zhang et al., 2020).

#### Conservational analysis of *IQSEC2*

Functional sites of proteins are more conserved than other parts of their surface. Therefore, residues with high conservation scores may be located in the functional region of a protein (Jackson et al., 2019). UGENE and T-coffee showed the degree of conversation and the multiple alignments of the IQSEC2 protein (Wang et al., 2021). Mutation Taster (https://www.mutationtaster.org/) is a web server that calculates the values of phylop and phastCons (Taghdiri et al., 2017). A positive phylop value is considered to be conservative, the larger the number, the more conservative the protein is, whereas a negative value is an opposite. PhastCons is reported as a value of 0-1, and the closer it is to 1, the more conservative it is.

#### Side chain properties of mutant amino acids and protein-protein interactions

Varsite is a web server (https://www.ebi.ac.uk/thornton-srv/databases/VarSite) that extracts information from UniProt clinvar, as well as gnomad, onto protein 3D structures in the PDB. It can evaluate whether missense mutations are pathogenic and provide annotations on the secondary structure of variant proteins, interactions with ligands, metals, DNA / RNA, or other proteins (Laskowski et al., 2020).

mCSM-PPI2 (http://biosig.unimelb.edu.au/mcsm_ppi2/), a machine learning computational tool designed to predict the effects of missense mutations on protein-protein interaction binding affinity (Rodrigues et al., 2019).

## Results

### Clinical features

Patient 1 was a 5-year-old male child with severe developmental delay and epilepsy. He showed an obvious ID. At the time of this study's evaluation, he could not walk independently. His language ability was worse than his peers, and he could only say a few words involuntarily. Seizures manifested as spasms, first appearing at the age of 4.5. Sodium valproate and levetiracetam were prescribed; however, these medications did not control the patient's seizures. Epileptiform discharges were detected by EEG, and no definite abnormalities were observed by MRI. He had a typical family history.

Patient 2 was a male patient aged 1 year and 6 months. His clinical manifestation was a global developmental delay. He could not stand alone and had no language development. His reaction to the outside world was deplorable, which manifested in his inability to look at people, communicate in non-verbal ways, or be made to laugh. There were no apparent abnormalities in EEG and MRI findings. His mother had mild ID but no seizures.

Patient 3 was a 4-year-old male child with global developmental delay and epilepsy. At the age of 2 years, he could walk independently; at the time of evaluation for this study, however, he had an obvious ID and no language development. He developed seizures at the age of 2 years, presenting with generalized tonic-clonic seizures. He has been treated with sodium valproate, and topiramate yet continues to experience seizures. A new form of seizure, namely, absence seizure, emerged. Apparent epileptic discharges were observed by EEG, and cranial MRI indicated poor myelination. There was no abnormality in the family history.

Patient 4 was a male child aged 2 years and 6 months with serious developmental delay and epilepsy. He could walk independently but fell quickly, and he could only say a few words involuntarily. In addition, he had the clinical manifestations of autism, including stereotyped behaviors such as putting his hand into his mouth, poor communication skills, and the inability to complete command behavior. He had experienced a generalized tonic-clonic seizure at the age of 2 years but had not been treated with any anti-epileptic drug. There were no abnormal findings in the MRI and EEG. Neither of his parents had any similar symptoms.

Patient 5 was a male patient aged 3 years and 6 months with severe developmental delay and epilepsy. At the time of evaluation for this study, he could walk independently without language development and had an obvious ID. He had poor responses to external stimuli and could not communicate with people in non-verbal ways, showing autism. Tonic seizures occurred at the age of 3 months. Valproate, topiramate, and other anti-seizure medications were prescribed to control these seizures; however, epileptic spasms continued 1 month later. Epileptic discharge was visualized on EEG, and cranial MRI showed bilateral ventricle enlargement. His parents were healthy and had no intellectual disability or epilepsy.

Patient 6 was a male child aged 2 years and 6 months with developmental delay and seizures. He could walk and run independently. He had an intellectual disability and no language development. He developed febrile seizures at 9 months but was not treated with anti-seizure medications. A generalized tonic-clonic seizure occurred at the age of 1 year, and levetiracetam was prescribed at the age of 2 years. No seizures had been reported for half a year. He had healthy parents.

**Figure 1:**
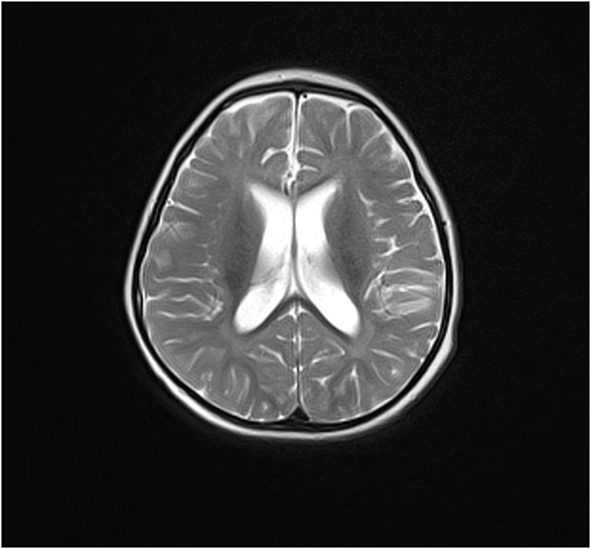
Transverse magnetic resonance (MR) images of patient 7. T2-blade shows delayed myelination and enlargement of the lateral ventricles.

Lastly, patient 7 was a 2-year-old male child with severe developmental delay and epilepsy. He could stand alone but did not have the ability to walk or speak. Epileptic spasms occurred at the age of 1 year and 7 months. The frequency of spasms decreased following adrenocorticotropin (ACTH) treatment; however, his condition was not completely controlled. His MRI finding showed two noticeable abnormalities in Figure 1. His parents were healthy without IDs or epilepsy (Table 1).

**Table 1:** Genotypes and phenotypes of patients with *IQSEC2* mutations.

**No**.	**P1**	**P2**	**P3**	**P4**	**P5**	**P6**	**P7**
Reported or not	Unreported	Reported	Unreported	Reported	Unreported	Reported	Unreported
Age (years)	5	1.5	4	1.5	3.5	2.5	2
Gender	Male	Male	Male	Male	Male	Male	Male
Genetic defect	c.316C > T	c.1849delC	c.443_4 44dup	c.804del	c.3235T > C	c.1075C > T	c.1417G > T
Protein defect	p.Gln106*	p.Arg617Ala fs*15	p.Ala149Glnf s*58	p.Tyr269Thrf s*3	p.Ser1079Pro	p.Arg359Cys	p.Glu473*
Variation type	Nonsense	Frameshifts	Frameshifts	Frameshifts	Missense	Missense	Nonsense
Truncated aberrant protein	–	+	+	+	–	–	+
Domain	NA	NA	NA	NA	Pleckstrin homology	IQ calmodulin–binding motif	NA
Origin	*De novo*	Mother	Mother	*De novo*	*De novo*	*De novo*	*De novo*
ACMG	Pathogenic	Pathogenic	Likely Pathogenic	Pathogenic	Likely pathogenic	Likely pathogenic	Likely pathogenic
Seizure	+	–	+	+	+	+	+
Age of seizure onset	4 years and 6 months	NA	2 years	2.5 years	3 months	9 months	1 year and 7 months
Seizure type	Spasms, atonic	NA	Absence, GTC	GTC	Spasms	FS, GTC	Spasms
Strabismus	–	+	–	–	–	–	+
EEG	+	–	+	–	+	+	+
Stereotypic movements	–	–	–	+	–	–	–
Delays in milestones	+	+	+	+	+	+	+
Dystonia	+	–	–	–	–	–	+
Intellectual disability	+	+	+	+	+	+	+
Speech dysfunction	+	+	+	+	+	+	+
ASD	–	+	–	–	+	–	–
MRI	–	–	Delayed myelination	Enlargement of the lateral ventricles	–	–	Delayed myelination and Enlargement of the lateral ventricles

ASD, autism spectrum disorder; FS, febrile seizure; GTC, generalized tonic-clonic seizures; NA, not available. The * symbol indicates termination.

### Sequencing analysis of trio-WES tests

**Figure 2:**
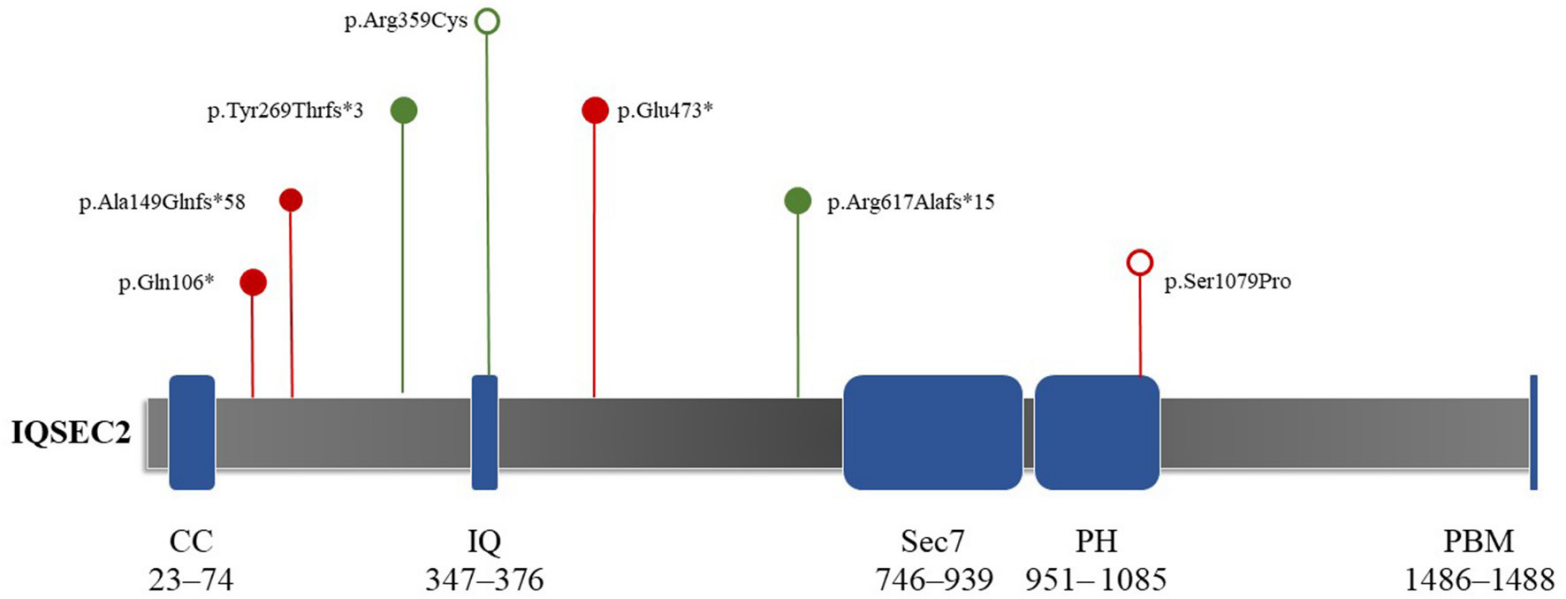
Schematic depictions of the domain structures of the protein encoded by *IQSEC2*. The protein length is 1,488 amino acids, covering five domains. The mutation locations in the seven study participants are shown. Red and green indicate reported and novel mutations in this study, respectively. Hollow circles represent mutations that do not truncate proteins (e.g., missense mutations). Solid circles show mutations that can truncate proteins (e.g., nonsense mutations and frameshifts). Both *de novo* missense mutations (c.3235T > C, p.Ser1079Pro; c.1075C > T, p.Arg359Cys) were located in the IQ and PH domains, respectively. CC, coiled–coiled (red); IQ, IQ-like motif (yellow); Sec7, Sec7 enzyme domain (green); PH, pleckstrin homology domain (gray); PBM, PDZ binding motif (orange).

Patient 1 showed a *de novo* nonsense mutation (c.316C > T, p.Gln106Ter) outside the domain. Patient 2 showed a frameshift mutation (c.1849delC, p.Arg617Alafs^*^15), inherited from his mother outside the domain. Patient 3 showed a frameshift mutation (c.443_444dup, p.Ala149GlnfsTer^*^58), inherited from his mother outside the domain. Patient 4 showed a *de novo* frameshift mutation (c.804del, p.Tyr269ThrfsTer^*^3) outside the domain. Patient 5 showed a *de novo* missense mutation (c.3235T > C, p.Ser1079Pro) local to the Pleckstrin homology (PH) domain. Patient 6 showed a *de novo* missense mutation (c.1075C > T, p.Arg359Cys) local to the IQ calmodulin-binding motif domain. Finally, patient 7 showed a *de novo* nonsense mutation (c.1417G > T, p.Glu473^*^) outside of the domain (Figure 2).

### Bioinformatic analyses

#### Pathogenicity and deleterious analysis

According to PolyPhen-2, both of the variants were identified as probably damaging. SIFT predicted the deleterious mutations, and scores are shown in Table 2. MutPred provided the molecular mechanism of protein functional change, such as an altered coiled-coil or loss of intrinsic disorder, and gave the probability of prediction with a *P*-value <0.05. As presented in Table 2, the values provided by CADD prediction were 29.7 and 26.5. It was believed that both missense mutations were disease-causing. The results reflecting a tendency were inconsistent because the software was based on different algorithms. In conclusion, we believed that both missense variants (c.3235T > C, p.Ser1079Pro; c.1075C > T, p.Arg359Cys) were deleterious or probably damaging.

**Table 2:** Prediction of 2 missense mutations identified to be deleterious or benign using different algorithms.

**Prediction method**		**Amino acid mutation site**	
		**p.Arg359Cys**	**p.Ser1079Pro**
CADD		29.7	26.5
SIFT	Score	0	0
	Prediction	Deleterious	Deleterious
PolyPhen-2	Score	0.997	0.957
	Prediction	Probably damaging	Probably damaging
MutPred2	Score	0.844	0.918
	Molecular mechanism (probability)	Altered coiled coil (0.09) Loss of intrinsic disorder (0.44) Altered DNA binding (0.14) Altered disordered interface (0.59)	Altered coiled coil (0.11)

#### Protein stability analysis

When using I-STABLE, amino acid substitutions S1079P and R359C were recorded as increasing the stability of the IQSEC2 protein. In Table 3, it can be seen that decreasing the strength of IQSEC2 protein in two mutations was tested by MUPro. Although the protein stability changed, the direction was different in the same mutations. The website PDB did not offer complete residue sequence information for IQSEC2. Thus, only S1079P could be predicted by DUET and CUPSAT. The missense variant (c.3235T > C, p.Ser1079Pro) was regarded as destabilizing the IQSEC2 protein in DUET. CUPSAT predicted that mutations did not lead to changes in protein stability. The DDG scores and change directions of the two *de novo* missense mutations (c.3235T > C, p.Ser1079Pro; c.1075C > T, p.Arg359Cys) on the stability of the IQSEC2 protein varied in terms of prediction, but most outcomes supported the impact of amino acid substitutions on protein stability.

**Table 3:** Four tools to evaluate the effect of amino acid changes on protein stability.

	**Prediction tool**	**Amino acid mutation site**	
		**p.Arg359Cys**	**p.Ser1079Pro**
ΔΔG (kcal/mol)	I–STABLE	0.61	0.74
	MUPro	−0.14	−1.21
	DUET	NA	−0.803
	CUPSAT	NA	0.07
Stability	I–STABLE	Increase	Increase
	MUPro	Decrease	Decrease
	DUET	NA	Destabilizing
	CUPSAT	NA	Stabilizing

NA, not available.

#### Conservational analysis of IQSEC2

**Figure 3:**
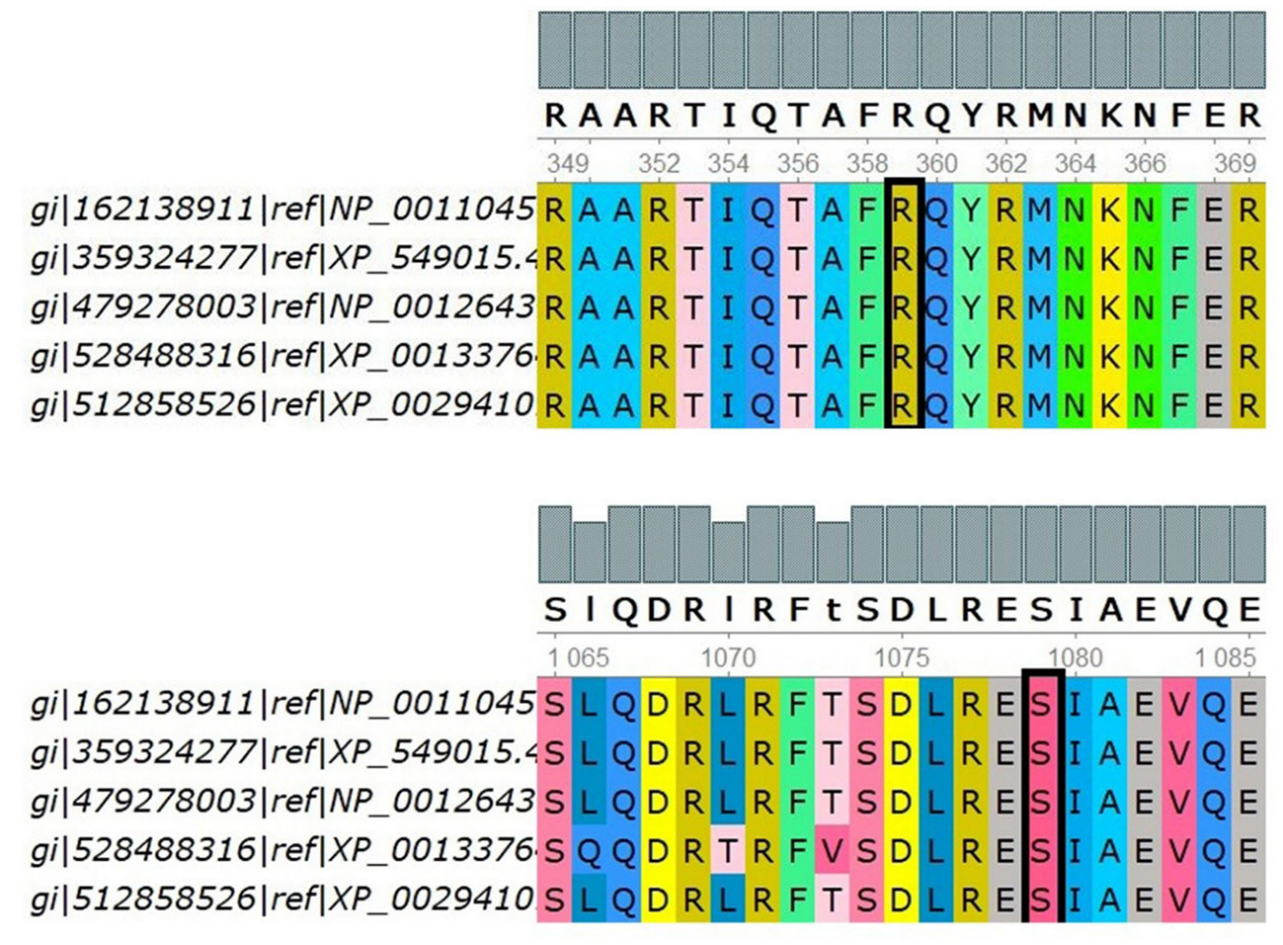
Multiple sequence alignments of the indicated IQSEC2 sequences using UGENE. The target site is selected by the black block, i.e., p.Ser1079 and p.Arg359. Five species were used to compare multiple sequence alignments, including H.sapiens, B.taurus, R.norvegicus, D.rerio, and X.tropicalis, by UGENE.

Positive phylop values (2.771 and 0.44) were found, respectively, and the PhastCons values were both 1, which indicated that Ser1079 and Arg359 were conservative. In Figure 3, multiple sequence alignments showed that Ser1079 and Arg359 of the IQSEC2 protein had strong evolutionary conservation among different species. It can be inferred that the residues Ser and Arg are functional sites in the IQSEC2 protein, and their change may affect the protein's function.

#### Side chain properties of mutant amino acids and protein-protein interactions

**Figure 4:**
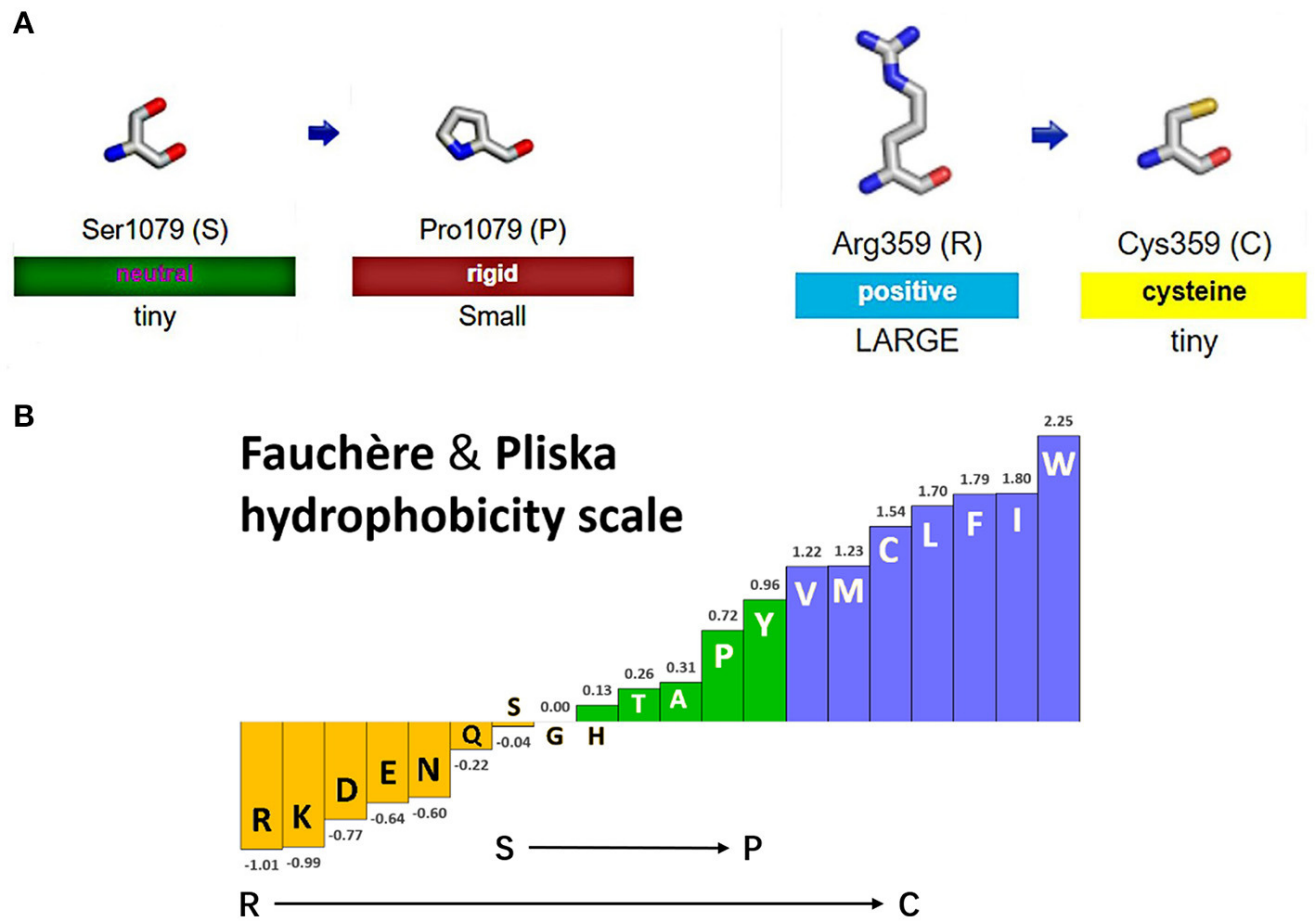
Display of side chain properties and hydrophobicity after missense mutations in IQSEC2 protein. **(A)** A schematic diagram of two missense variants. **(B)** Fauchère and Pliska hydrophobicity scale exhibited the hydrophobicity of 20 amino acids. Abscissa: from left to right, hydrophobicity gradually increased. Hydrophobicity changes of amino acid substitution were shown.

Figure 4A shows the change of side chains. The residue at sequence position 359 in this protein was an arginine with a positively charged side chain, making it hydrophilic. The variant residue was a cysteine with a side chain capable of forming a disulfide bond with another cysteine, providing solid structural support for the protein. We could see a large positive side chain turn into a tiny cysteine residue which was a considerable change and might result in a shift in the protein's function. The residue at sequence position 1,079 in this protein was a serine with a neutral side chain. The variant residue was a proline with a rigid side chain restricting the conformation of the protein at this point. A change from a Ser to a Pro side chain was not large and may or may not result in a shift in the protein's function. The relative hydrophobicities of the 20 amino acids based on the Fauchère and Pliska hydrophobicity scale are shown in Figure 4B. The hydrophobicity of the R359C variant was a significant change, as the side chain of Arg was highly hydrophilic, while the Cys residue was hydrophobic. The change of S1079P was not substantial.

**Figure 5:**
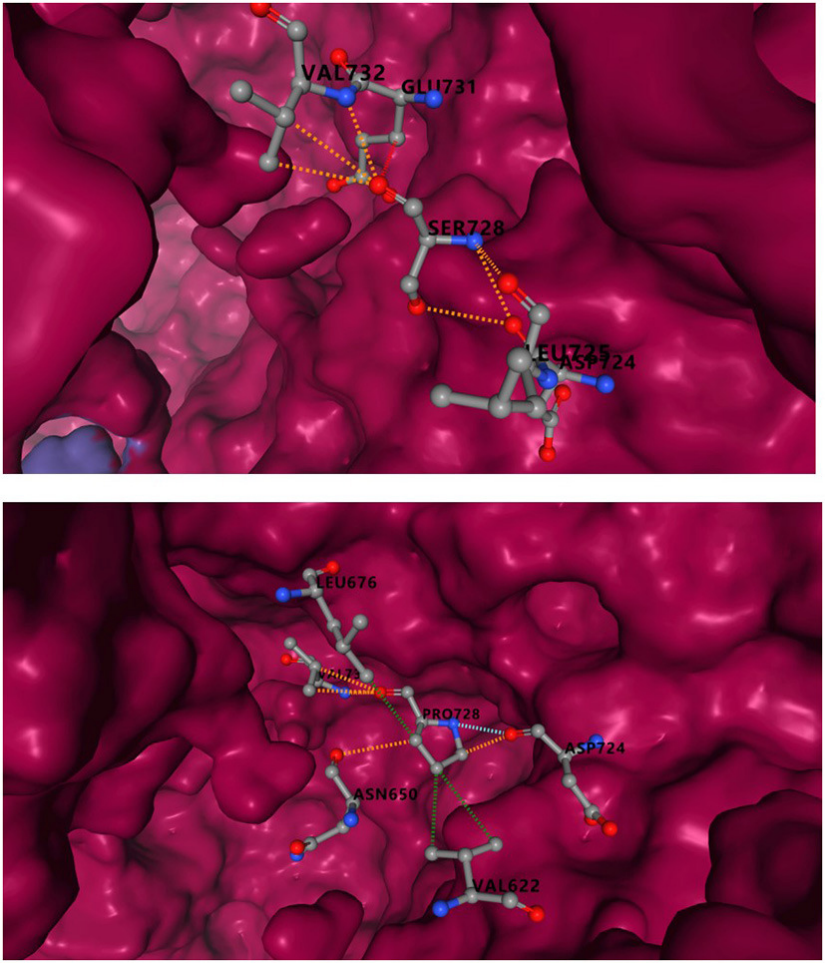
Inter-residue interactions in S1079P variant. Different colors represent different interactions. Blue is van der Waals (VDW), red is hydrogen bond, green is hydrophobic, and orange is polar. The interaction between serine and other residues is shown at the top, and the interaction between proline and other amino acids is shown at the bottom.

Incomplete structure of IQSEC2 protein in PDB database, the amino acids sequence 729-1099 could be obtained, and the variant R359C was excluded. Ser in S1079p site corresponded to 728 in PDB. The predicted results showed that serine substitution for proline resulted in a decrease in binding affinity of 0.459kcal/mol. In Figure 5, mCSM-PPI2 simulated the effects of S1079P mutation on structural alteration and interactions with other amino acids. Hydrogen bonds were formed with VAL732, GLU731, and ASP724 in wild-type. After mutation, the hydrogen bonds between GLU731 disappeared, and ASN650 was built. Compared with the wild-type, the mutant was provided with hydrophobicity, the van der Waals force added between ASP 724, and the polar also changed.

### Effect of 7 mutations

All mutations were explored within databases. Mutations in Patients 1, 2, 3, 4, and 7 led to protein truncation; therefore, they were likely pathogenic according to the ACMG. The other two mutations classified as pathogenic were assessed using mutation prediction tools. Subsequently, the clinical implications and considerations for evaluating *in silico* algorithms were investigated according to the clinical variant interpretation guidelines established by the ACMG.

## Discussion

*IQSEC2* encodes GEFs for the ARF family of GTP-binding proteins (Shoubridge et al., 2019). Multiple *IQSEC2* mutations have been reported (Jackson et al., 2019; Accogli et al., 2020; Brant et al., 2021) and may lead to global severe developmental delay and epilepsy. Our study found four novel *IQSEC2* mutations related to overall developmental delay and seizures.

Previous studies have reported that the type of mutation is related to the severity of *IQSEC2*-related diseases. Prior research suggests that mutations leading to protein truncation are more likely than missense mutations to cause serious phenotypes (Gandomi et al., 2014), but this conclusion was not supported in this study. Pathogenicity, protein stability, protein conservation, side chain properties and protein-protein interactions after single amino acid variants were analyzed in a variety of ways for patients 5 and 6, who had missense mutations, and revealed that both missense variants (c.3235T > C, p.Ser1079Pro; c.1075C > T, p.Arg359Cys) were deleterious or probably damaging, located in the functional sites, affected protein stability, and changed the side chain properties and protein affinity. These results indicated that missense mutations impacted the structure and function of the IQSEC2 protein. In the clinic, the mutation in patient 2 may lead to protein truncation, but the patient showed no severe symptoms such as epilepsy or repeated and stereotyped behavior. In contrast, patients 5 and 6 showed early-onset epilepsy, especially in patient 5, who exhibited refractory epilepsy and infantile epileptic spasms syndrome. Their mutation type was a missense mutation, which cannot lead to protein truncation; however, their mutation was located in the key domain (IQ and PH). The findings of this study were consistent with those of Zerem et al. (2016), where three out of nine patients with epileptic encephalopathy had missense mutations located in essential domains (IQ-like or Sec7).

*IQSEC2* is expressed in neurons and affects the cytoskeletal organization, excitatory synapses, and dendritic morphology formation (Kalscheuer et al., 2015; Hinze et al., 2017). It can encode GEFs for the ARF family of GTP-binding proteins (Shoubridge et al., 2019). There are five domains and 1,488 amino acids in the encoded protein, including a coiled–coiled (CC) domain (23–74 amino acids), IQ-like motif (347–376 amino acids), Sec7 domain (746–939 amino acids), PH domain (951– 1085 amino acids), and PDZ binding motif (1486–1488 aa) on the C′ end (Figure 2) (Shoubridge et al., 2010b; Kalscheuer et al., 2015; Wayhelova et al., 2020). The PH domain attracts proteins to membranes *via* interactions with phosphoinositide and guides the ArfGEF to appropriate cellular compartments (Feng et al., 2019). Shoubridge et al. (2022) showed that missense mutations in the PH domain result in severe phenotypes in male individuals, including severe intellectual disability, epileptic encephalopathy, and autism. In this study, patient 5 had a missense mutation in the PH domain. His main phenotype was infantile epileptic spasm syndrome, which appeared in the early stage of his life. Notably, this result matched the outcomes of Shoubridge et al. (2022). Our findings also support the conclusion that missense mutations in the PH domain can lead to severe symptomatic phenotypes.

A frameshift mutation in patient 3 was found, inherited from his mother. This mutation causes a truncated aberrant protein. Interestingly, no corresponding symptoms were found in the proband's mother. Wayhelova et al. (2020) identified a novel frameshift variant (c.1813_1814del, p. Asp605Profs^*^3) in *IQSEC2* in both of their patients and the patients' unaffected mothers who had skewed X chromosome inactivation (XCI) (100:0). We evaluated the mother of patient 3. She showed an extreme skewness ratio of 100:0, with skewed inactivation of the X chromosome carrying the mutated *IQSEC2* gene. These hypotheses require further clinical validation.

## Conclusion

We reported four novel *IQSEC2* mutations in this paper. Severe phenotypes, such as seizures in early childhood, usually occur in male patients with *IQSEC2* mutations, especially mutations in the PH and Sec domains. Some female *IQSEC2* mutation carriers are asymptomatic, which is attributed to the skewed inactivation of the X chromosome. This conclusion suggests that a genetic diagnosis of *IQSEC2* mutations is necessary for children with global developmental delay and seizures.

## Data availability statement

The data that support the findings of this study are available from the big data center of Chinese People's Liberation Army General Hospital, but restrictions apply to the availability of these data and therefore are not publicly available. Data are however available from the authors upon reasonable request and with permission of the Chinese People's Liberation Army General Hospital.

## Ethics statement

This study was reviewed and approved by the ethics committee of the First Medical Center of the PLA General Hospital. Written informed consent was obtained from the minor(s)' legal guardian/next of kin for the publication of any potentially identifiable images or data included in this article.

## Author contributions

GY contributed to the conception and design of the study. LW and XL organized the database and wrote the first draft of the manuscript. All authors contributed to manuscript revision, read, and approved the submitted version.

## Funding

This research was partially supported by the Natural Science Foundation of Beijing, China (No. 7222187), the Medical Big Data and Artificial Intelligence Research and Development Project of the Chinese PLA General Hospital (No. 2019MBD-004), the Epilepsy Research Fund of China Association Against Epilepsy (No. CU-B-2021-11), and the Nutrition and Care of Maternal & Child Research Fund Project of Guangzhou Biostime Institute of Nutrition & Care (No. 2021BINCMCF030).

## Conflict of interest

The authors declare that the research was conducted in the absence of any commercial or financial relationships that could be construed as a potential conflict of interest.

## Publisher's note

All claims expressed in this article are solely those of the authors and do not necessarily represent those of their affiliated organizations, or those of the publisher, the editors and the reviewers. Any product that may be evaluated in this article, or claim that may be made by its manufacturer, is not guaranteed or endorsed by the publisher.
